# 3D-Printed Products for Topical Skin Applications: From Personalized Dressings to Drug Delivery

**DOI:** 10.3390/pharmaceutics13111946

**Published:** 2021-11-17

**Authors:** Rafaela Santos de Oliveira, Stephani Silva Fantaus, Antonio José Guillot, Ana Melero, Ruy Carlos Ruver Beck

**Affiliations:** 1 Programa de Pós-Graduação em Ciências Farmacêuticas, Universidade Federal do Rio Grande do Sul. Avenida Ipiranga, 2752, Porto Alegre 90610-000, Brazil; oliveira.rafaela@ufrgs.br; 2 Departamento de Produção e Controle de Medicamentos, Universidade Federal do Rio Grande do Sul. Avenida Ipiranga, 2752, Porto Alegre 90610-000, Brazil; stephanifantaus@gmail.com; 3 Department of Pharmacy and Pharmaceutical Technology and Parasitology, School of Pharmacy, University of Valencia, Avenida Vicente Andres Estelles SN, 46100 Burjassot, Spain; antonio.guillot@uv.es

**Keywords:** additive manufacturing, wound dressing, FDM, skin disorders, film, patch, 3D-printing

## Abstract

3D printing has been widely used for the personalization of therapies and on-demand production of complex pharmaceutical forms. Recently, 3D printing has been explored as a tool for the development of topical dosage forms and wound dressings. Thus, this review aims to present advances related to the use of 3D printing for the development of pharmaceutical and biomedical products for topical skin applications, covering plain dressing and products for the delivery of active ingredients to the skin. Based on the data acquired, the important growth in the number of publications over the last years confirms its interest. The semisolid extrusion technique has been the most reported one, probably because it allows the use of a broad range of polymers, creating the most diverse therapeutic approaches. 3D printing has been an excellent field for customizing dressings, according to individual needs. Studies discussed here imply the use of metals, nanoparticles, drugs, natural compounds and proteins and peptides for the treatment of wound healing, acne, pain relief, and anti-wrinkle, among others. The confluence of 3D printing and topical applications has undeniable advantages, and we would like to encourage the research groups to explore this field to improve the patient’s life quality, adherence and treatment efficacy.

## 1. Introduction

Skin is the largest organ in the human body and plays an important role in emotional and physical welfare [1,2]. The skin covers the whole-body surface, being the first defense against xenobiotic entrance [3]. Due to its extension, it has been considered as a drug application site for local, but also for systemic aims. It is widely known that certain substances can passively diffuse through the skin and produce both, local and, less commonly, systemic effects, but this feature is limited to a few drugs [4].

Skin diseases are a problem for the world population as one-third of the population is affected by these disorders [5]. Still, skin diseases are the fourth most common cause of human infirmity and represent a major problem in the United States healthcare system [5,6]. Moreover, wound treatment, especially chronic wounds, represents an important health care burden, with global care costs varying from $13 to $15 billion annually [7]. Consequently, novel technologies for the treatment of skin diseases and injuries are highly encouraged.

Nowadays, topical products are a lot more complex, varying from simple solutions and creams to multiphase, nanotechnology and assisted technologies [4]. In this scenario, 3D printing is a promising alternative to develop and produce biomedical and pharmaceutical products aiming at the treatment of skin disorders and skin injuries. This technology allows the design and control of specific desired properties, porosity, pore size, roughness, functional groups, size, shape, swelling, degradability profile, drug loading and drug release profile [8,9,10]. Fundamentally, these properties can provide similarities to the skin’s native environment and provide better conditions for diseases/injury treatment.

3D printing has emerged in the past years as an innovative and versatile platform for the development of new healthcare solutions. Although it is a well-consolidated technology in other industries, such as automotive and aeronautics, in the pharmaceutical and biomedical fields it is still new and with a promising path ahead [8]. The first drug produced by 3D printing and approved by the Food and Drug Administration (FDA) was only in 2015, highlighting its innovative potential [11]

3D printing has been well explored in oral drug delivery to produce the most diverse drug release profiles and customizations [12]. Different approaches have been proposed for controlled [13], sustained [14], or fast [15] release of drugs and for the production of the so-called polypills with different drug release profiles [16]. Orodispersible films, chewing tables, and implants are just some other pharmaceutical dosage forms that have been explored by the wide range of 3D printing possibilities [12,17].

Regarding skin applications, the use of 3D printing tools for improving skin drug delivery with systemic and local applications is an emerging area of research, which is in exponential growth. 3D printing has been recently used to produce microneedles, aiming, mainly, for transdermal applications [18]. Moreover, with the improvement of the printing techniques and, therefore, with cell bioprinting, e.g., the printing of cells, these systems have been intensively explored for skin tissue engineering [19]. Yet, some disadvantages can be cited, as the use of cells relies on strict regulatory control [19,20]. Despite all these skin applications, 3D printing for topically applied local drug delivery systems onto the skin and wound dressing without cells is still a modest but highly promising research field.

There are excellent reviews that cover 3D printing and its applications on the skin, as transdermal drug delivery [21] and bioprinting [22,23,24]. However, to the best of our knowledge, none of them were dedicated to discussing the association of 3D printing and pharmaceutical topical skin products for drug delivery and dressings. In this review, we mainly focus on the applications of 3D printing in topical drug delivery with a local therapeutic aim and for plain dressing. Accordingly, this work provides an updated review of the aforementioned association in the treatment or prevention of skin diseases. Therefore, we first discuss the topical route and main 3D printing techniques applied to this field and thereafter, based on the literature research performed, a discussion of the studies that comprise 3D printing and topical skin application. Lastly, challenges and perspectives are discussed, followed by a brief conclusion.

## 2. Topical Delivery

Topical administration refers to the application of formulations on the superficial area of the skin for the local treatment of diseases or injuries [25]. Site-specific drug delivery can target several skin sites, such as stratum corneum (SC), viable epidermis, dermis, pilosebaceous unit, hypodermis and deeper tissues [26]. The main advantages of this delivery route are patient acceptance; its painless and noninvasive properties; improvement in drug skin bioavailability; better pharmacological and physiological response; and minimum systemic toxicity and drug exposure to non-desired sites [25]. In this article, we will focus on localized skin active ingredient delivery, which implies targeting specific skin layers, while reducing systemic absorption.

Skin active ingredient delivery requires a complex understanding of the skin structure, physiology, skin disease, drug, and vehicle characteristics. There are some intrinsic skin factors that can affect active ingredient delivery across the skin, such as the skin barrier function, provided mainly by the stratum corneum, skin hydration, anatomical site, pathological effects, age, ethnicity and gender, among others [27]. Hydration is also an important factor that influences penetration, as well hydrated areas provide better permeation conditions [28].

**Figure 1:**
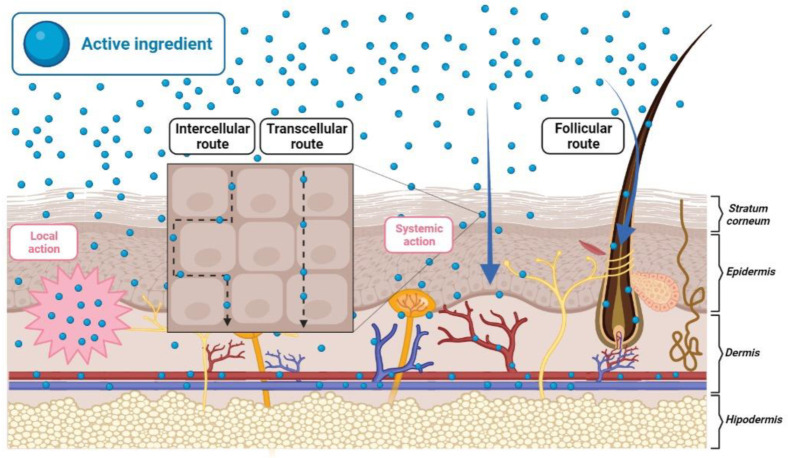
Skin layers and drug delivery routes for systemic and local action.

The therapeutic effect of the topically applied formulations relies on the following three steps: i. Release of the active ingredient from the formulation/dosage form; ii. Partition and distribution of the drug within the skin barrier, the SC, and permeation/diffusion through the SC; iii. Partitioning and diffusion from the SC to the viable epidermis and/or dermis, where the active ingredient should produce the desired pharmacological effect [29,30]. The passage of drugs into the skin or through the skin involves three potential routes (Figure 1): the transcellular route, which is related to the drug transport through the lipid matrix and corneocytes of the SC; the intercellular route, in which the drug diffuses between the lipid matrix and the corneocytes; and the transappendageal route, in which the drug is transported through the hair follicles, sebaceous glands or sweat glands [26,31].

The vehicle containing the active ingredient and its characteristics, such as formulation type (e.g., emulsions; gels; alcoholic, hydrophilic, or lipophilic properties), concentration of the components, presence of permeation enhancers (surfactants and chemicals that can interact with the stratum corneum and make it more permeable), pH, viscosity, and charge can also importantly influence drug delivery rates and their permeability [27,31]. In addition, occlusive formulations will always improve drug permeability through an increase in skin hydration [27]. Therefore, when designing the ideal formulation, it is important to understand all the variables affecting the active ingredient penetration and permeation across the skin [32]. The vehicle should also be designed and manufactured according to the disease and skin area to be treated [33,34].

Nowadays, semisolid formulations are preferred for dermatological use. Powders and solutions show important disadvantages for topical drug delivery, such as low retention time on the skin, thus leading to a transient drug release and therapeutic effect. Semisolid, or even solid, formulations provide a more adequate permanence of the active ingredient onto the skin and are able to prolong its release. Therefore, different types of pharmaceutical formulations have been developed and produced to be applied onto the skin, such as ointments, emulsions, gels, patches, films, among others [35].

One of the major drawbacks in active ingredient skin delivery is the barrier function of the SC. To effectively diffuse through the skin, molecules must fulfill a set of requirements, such as being small molecules (<~500 Da), an adequate partition coefficient (log 1–3) and ionization degree [36,37]. Unfortunately, a great range of pharmaceutically interesting molecules does not fulfill these criteria, such as peptides, proteins and vaccines, making the delivery across the skin a difficult process [36].

Some approaches have been proposed to increase site-specific drug delivery, such as including penetration enhancers, use of supersaturated systems, prodrugs, nanocarriers, physical and mechanical methods (e.g., microneedles, abrasion, stretching) and electrical methods (e.g., iontophoresis, electroporation) [38]. Some of them will be discussed further in this review.

In addition to the use of drug-loaded devices, sometimes it is necessary to use non-medicated dressings for wound healing processes. In this case, more complex dressings, that can offer ideal conditions for the wound healing process, are being explored [39]. Importantly, these dressings can be individually customized to preserve patient anatomy and needs, thus ensuring good efficacy and acceptable aesthetic aspects.

The ideal wound dressing should provide some features, such as mechanical protection of the wound; protection against infections and microorganisms; oxygen permeability; control of the humid environment; excess exudate removing; be easily peeled off from the wound; biodegradable, biocompatible; non-toxic; and cost-acceptable [39,40]. Nonmedical dressing can be composed of a wide range of materials, as hydrocolloids, alginates, chitosan and hydrogels, among others [39,41].

The current trend is to replace traditional dressings, such as cotton or gauze, which do not have any therapeutic effect, with technological and individualized dressings, providing a more personalized therapy [12,41]. Over the course, the wound dressing can be divided into three categories: bioactive; passive or inert; and interactive [42]. Bioactive dressings are characterized by the gradual release of bioactive molecules. Inert or passive dressing are common materials that provide covering and protection of the wound against pathogens from the external environment, acting mainly as a physical barrier. Interactive wound dressing can influence the wound surface and afford benefits to the healing process. They are able to influence the wound environment and can reduce exudates, prevent infections, form granulation tissue and re-epithelialize and even facilitate debris removal [42,43]. Another classification used relies on their complexity, divided into traditional dressings (e.g., gauze), modern (e.g., films and foams) and advanced dressings (e.g., medicated modern dressing and tissue-engineered substitutes) [44,45].

Some of the commercially available skin dressings include TegadermT™, DermaView II™, DuoDERM^®^, ALGISITE^◊^ M, Exuderm™ LP, Tegasorb^MR^, AquaFlo™, etc., and they vary from films, hydrogels, hydrocolloids, etc., [46]. Although these commercial dressings hold great properties and advantages, some of them still present limitations, such as high cost and low adhesion to the lesion surface, which may lead to additional injury upon replacement of the dressing [47]. In this review, we will discuss wound dressing and how 3D printing can benefit this therapeutic approach.

## 3. 3D Printing Techniques

Three-dimensional printing (3D-printing) is an additive manufacturing method that allows the production of objects layer-by-layer based on a digital file, using computer-aided design (CAD) or imaging techniques, such as magnetic resonance imaging or scan [48,49,50]. The digital file is converted to stereolithography (.stl) file format, which is interpreted by the printer software. Thus, the translation of a digital file into a physical object allows the manufacture of personalized and individualized objects [51].

3D printing was introduced to the market approximately three decades ago and has impacted significantly in many industrial fields. Nowadays, this technology benefits from the possibility of creating prototypes quickly and being cost-effective. The reality is that 3D printing is only limited by the power of imagination and the features of the available printers and techniques [50].

More recently, 3D printing has shown that it can be used in healthcare and that it has a high potential to improve existing therapies. In this area, it can be applied from printing personalized prostheses, implants, and cells to products containing drugs or nanomaterials [8,50]. Supporting the concept of personalized medicine, 3D printing came to disrupt some previously well-established concepts. There is a trend to complement the “one-size-fits-all” concept, promoted by large-scale manufacturers, going ahead also with the personalization of therapies. This is driven by the proposition that one dose may not suit everyone, as the dose can vary with weight, age, disease, and genetics [49,50]. In view of this, personalized therapy aims to meet the individual needs of each patient or group of patients, and thus promote better therapy.

Different 3D printing technologies are currently available for several applications [8]. The different 3D printing techniques are characterized by unique properties, regarding resolution, output volume, cost-efficiency, biocompatibility, among others, which can influence the final printing objective [52]. Therefore, 3D printing techniques can be divided into seven categories: material jetting, material extrusion, vat photopolymerization, powder bed fusion, binder jetting, sheet lamination and directed energy deposition [51]. As there are great reviews focusing on the 3D printing techniques for pharmaceutical applications, as fused deposition modeling (FDM) [53], semisolid extrusion (SSE) [10], inkjet printing [54], stereolithography [55], and powder bed fusion [56], here, only a brief overview of the most used techniques in the pharmaceutical field will be discussed.

**Figure 2:**
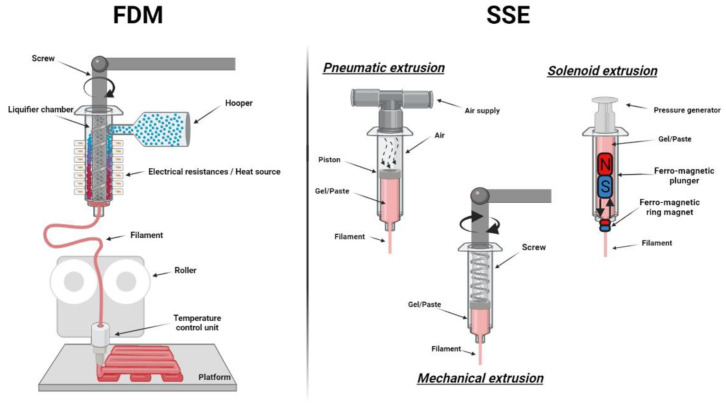
Extrusion based techniques, fused deposition modeling (FDM) (**left**) and semisolid extrusion (SSE) mechanisms (**right**).

The extrusion-based system includes mainly FDM and SSE (Figure 2) [57]. Among them, FDM is one of the most employed techniques in the pharmaceutical and biomedical fields, owing to its low cost and efficiency. This is a thermo-based technique, so thermoplastic materials are widely used, due to their low melting point, an important parameter in FDM. Solid polymeric filaments are placed in the printer, and by temperature control, it melts during extrusion. Subsequently, the material hardens before another layer is laid onto the previous one, to create a new structure [58]. Moreover, SSE is also largely used in the pharmaceutical and biomedical fields for the development of semisolid structures and formulations. SSE is also known as robocasting, cold extrusion-based printing, hydrogel-forming extrusion, melting extrusion, melting solidification printing process, thermal extrusion, direct ink writing, hot-melt pneumatic extrusion, melting extrusion, micro-extrusion, pressure assisted micro syringe (PAM). This technique relies on the extrusion and deposition of gels or pastes, being the nature of the material that differs from FDM and what makes it important also for bioprinting applications [10]. There are three main SSE mechanisms: pneumatic, mechanical, or solenoid-based systems, and they vary from the use of pressurized air to mechanical force and electrical pulse, respectively (Figure 2). While FDM requires high temperature, SSE can be used at low temperature. However, during SSE printing, a post-printing process is often required, as a drying process or cross-linking phase [10,57].

Laser techniques, as SLA and digital light processing (DLP), is based on photopolymerization that use light irradiation to build 3D solid structures. Compared to other technologies, these methods offer several advantages including greater feature resolution, a smoother surface finish and avoidance of drug thermal degradation [55,59]. Jetting techniques, such as inkjet, electrohydrodynamic jet (EHD) and binder jet printing, are also used in pharmaceutical approaches. The EHD technique was developed from electrospinning and provides layer-by-layer deposition of aligned fibers, while inkjet printing includes all systems that place small liquid drops on a substrate [60,61,62]. Binder jet printing is based on the agglomeration of powdered material, layer by layer [60] and has been reported as the most successful 3D printing technology since it was used to print Spritam, the first 3D printed medicine approved by the FDA [51].

In addition to the aforementioned, 3D printing can be applied in mold production of pharmaceutical dosage forms [63]. The solvent casting method has been used to prepare polymeric films for tissue engineering, drug delivery, or coating procedures, and is based on the deposition of polymeric solutions in a 3D-printed mold to create a structure with the negative shape of the mold after solvent evaporation [64].

Encouraged by the development of 3D printing technology, a great range of polymers are arising to achieve diverse requirements of biomedical applications [58]. Basically, polymers, ceramics and hard metals can be used [65]. Polymers are widely used in pharmaceutical dosage forms [12] and biodegradable scaffolds [66]. The polymers used in the biomedical field meet medical standards, such as biocompatibility and are very versatile, allowing their use in more than a single technique [67]. Understanding the rheology of the polymer and how drugs can affect printability is important for efficient 3D printing [57].

Natural polymers, i.e., chitosan, collagen and alginate are ideal for biomedical applications, even though they can have poor mechanical strength [68]. FDM has still limited materials suitable for printing due to the use of high temperatures during the process, and because thermoplastic polymers are preferred. The main polymers reported for this technique are PVA (poly(vinyl alcohol)), PLA (polylactic acid) and EVA (Ethylene Vinyl Acetate) [69]. Moreover, PCL (polycaprolactone) [70,71] and polymethacrylates, as Eudragit^®^ [72] are being well explored in the pharmaceutical field due to their versatility. On the other hand, some 3D printing technologies, i.e., SLA, lack a great range of materials available, once most of them are not recognized as safe (GRAS) [53]. In addition to the main polymers, other excipients can be necessary for successful 3D printing and adequate mechanical properties, such as plasticizer, antioxidant, insoluble or soluble filler, lubricant, among others [70,72,73,74].

## 4. Review Methodology

In this review, Web of Science, Pubmed and Scopus were used as databases for the literature search, up to 30 of September of 2021. The keywords used were: ((3D print*) and (skin or topical or dermal) and (drug* or scaffold* or film* or patch* or wound dressing or dressing* or mat or bandage)) and (3D print* and wound dressing). Only experimental articles that met the following inclusion criteria were included: use of 3D printing technology for pharmaceutical or biomedical products aiming topical skin applications. The exclusion criteria were transdermal (aiming systemic circulation); skin grafts; product implantation on the skin; bioprinting (cell printing); cell-laden scaffolds prior in vivo studies; articles that have not yet demonstrated practical topical applicability; review articles and access to the full text of the papers not available. Potential articles identified during the reading that fulfill the inclusion criteria were also added. Although the use of cell-laden scaffolds and bioprinting are the main topics found in the database research, 3D printing of products without cells for dermatological use seems to be still an emerging research area, but with great potential ahead. Applying the inclusion and exclusion criteria described above, 66 articles were comprised in this review.

## 5. 3D printing for Topical Skin Applications

According to the literature, 3D printing is a promising tool to develop personalized dressing and drug dosage forms in the manufacture of biomedical/pharmaceutical products for topical use.

**Figure 3:**
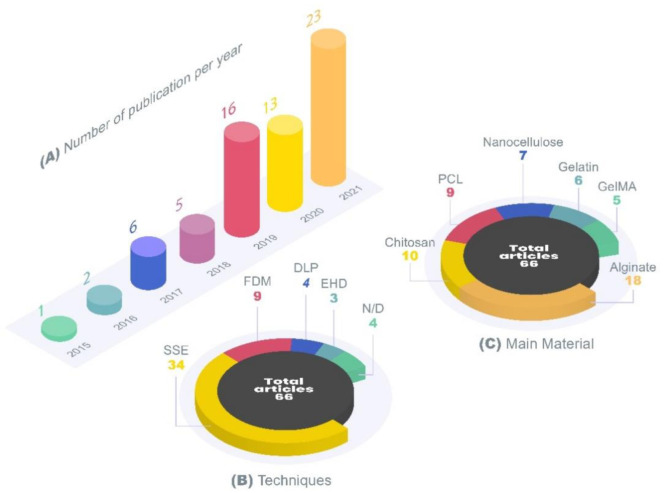
Main characteristics of the studies included in this review: (**A**) number of publications per year using 3D printing for topical skin applications; (**B**) number of records of the most employed 3D printing techniques; (**C**) number of records of the most employed materials; DLP, digital light processing; EHD, electrohydrodynamic jet; FDM, fused deposition modeling; GelMA, gelatin-methacryloyl; N/D, not clearly defined (according to the description described by the authors, it was not possible to categorize it in this review); PCL, polycaprolactone; SSE, semisolid extrusion.

An exponential growing interest in this field can be evidenced by the increasing number of published articles per year. Approximately 79% of the included articles were published in the last 3 years (2019, 2020 and 2021), highlighting the potential alliance between 3D printing and topical skin application (Figure 3).

Regarding the 3D printing techniques, extrusion-based were the most widely used technology, representing approximately 76% of the techniques applied. Among these techniques, SSE appears in an important number of articles published, indicating the relevance of this 3D printing method for skin applications. This technology holds great advantages in the use for the formulation of topical systems, as it allows the use of a great range of polymers. In addition, it is able to create the most diverse therapeutic approaches and does not need high-temperature ranges nor implies laser use. Additionally, SSE allows hydrogel dressings printing, which are systems with high water content and can be used to treat several skin conditions, such as acne, cellulite, burn wounds to mycosis and psoriasis [75]. They provide moisture retention, skin hydration and thus, an optimal environment for wound healing and the treatment of other skin diseases [75]. Another advantage is the possibility to easily load antimicrobials, biological signaling and pain relief molecules, among others, into these 3D-printed hydrogel systems [10,75]. Hydrogels are an interesting option for topical treatment, and their use in the production of 3D printed products comprised a significant portion of the studies included in this review.

Different printing techniques require different polymeric materials, leading to a high diversity of materials used to produce these 3D products. It is important to note that most articles used more than one material for the 3D printing and Figure 3C represents the number of records of the most reported materials. Alginate was the most used material, followed by chitosan and its derivatives, PCL, nanocellulose, gelatin and gelatin-methacryloyl (GelMA) (Figure 3). Alginate is a very versatile polymer and is already widely used in dressings on the market. It is a natural polymer that has good biocompatibility, biodegradability, swelling and absorption properties, and is also associated with promoting the healing process [76]. Throughout the article, some advantages and disadvantages of alginate, chitosan, PCL, gelatin and nanocellulose will be discussed.

**Figure 4:**
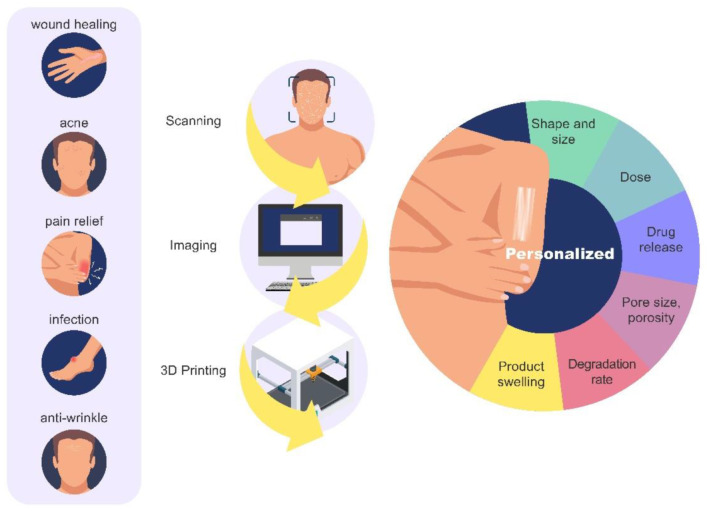
Main applications and advantages of 3D printing for topical skin products.

3D printing can be used for customizing dressings and patches, based on the individual anatomical needs of each patient, with the aid of virtual scanning. In addition, several properties can be targeted, ranging from porosity, fluid absorption, and degradation characteristics to more complex drug release profiles and dose characteristics. Several dermatological disorders can benefit from 3D printing, such as acne, infection, pain resolution, scarring, among others. Figure 4 summarized the main applications and advantages of 3D printing for topical skin products.

**Figure 5:**
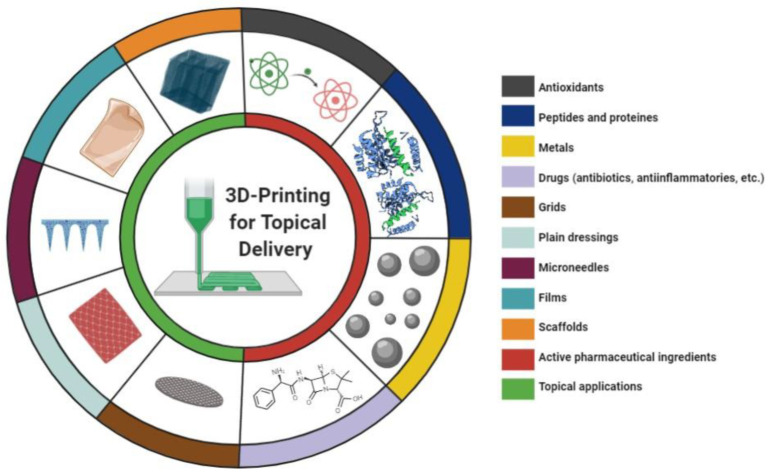
3D printed products (left side of the circle) and types of active ingredients (right side of the circle) previously proposed for topical skin applications.

Different topical 3D printed products and types of active ingredients were described in the studies (Figure 5), demonstrating the wide range of 3D printing possibilities for skin applications. Dermatological approaches to treat or prevent skin disorders may include the use of plain dressing for wound healing purposes or dosage forms containing active ingredients. Here, for an easy-to-read format, this paper was divided into two main topics: “Plain Dressings” and “Active Ingredients Delivery”.

### 5.1. Plain Dressings

Although the formulation of drug delivery systems has been an important strategy for the treatment of skin disorders, many wound healing treatments can be designed as non-medicated/plain dressing. 3D printing can address several limitations of conventional wound dressings by tailoring its mechanical and physical properties and providing a better wound healing environment. Most articles presented in this section used the SSE printing technique probably because of its aforementioned advantages. Based on the data acquired, alginate and nanocellulose are the most explored polymers for this purpose. In addition, hydrogel scaffolds seem to be the best alternatives for conventional wound dressing, due to their ability to keep a humid environment, allow oxygenation, promote proliferation, protect the injury from contamination and also because it can be easily removed [77,78]. In this scenario, this section will focus on passive and interactive wound dressing/skin substitutes, and on the discussion of how 3D printing can influence the development of this kind of skin product. Table 1 summarizes the articles regarding plain wound dressings.

Among the polymers used alone or in combination with other biocompatible polymers widely used for dressing applications, we will briefly discuss the most described and their main characteristics:

#### 5.1.1. Algae-Derived Biomaterials

Alginate is a biocompatible polymer that enhances cell proliferation and facilitates the absorption of wound exudates. It has been widely explored in the development of approaches to promote wound healing [76]. Despite its advantages, it also presents some drawbacks. Polysaccharide hydrogels can suffer from bad mechanical properties. Based on that, Milojevic and coworkers (2021) developed a hybrid 3D printed method by alternating the deposition of stiff thermoplastic polymer, PCL, and softer hydrogels, alginate/carboxymethyl cellulose. The hybrid scaffold demonstrated macroporous scaffolds with improved mechanical and physicochemical properties, which showed desired architecture. Compared to the hydrogel, the degradability of the hybrid scaffold was significantly delayed (from 2 days to more than 30 days), which is an important property to observe when designing wound dressings. Compared to PCL scaffolds without the hydrogel layer, the hybrid scaffolds showed higher swelling values, once the hydrogels add hydrophilic groups to the scaffolds. Mechanical strength values were between those shown by the scaffolds containing pure PCL and pure hydrogels, which could be attributed to their hybrid composition. Besides, the authors suggested that by adjusting the mass ratios of soft polymers and PCL a fine control of the mechanical properties could be provided, being an interesting approach for the development of personalized dressings [97].

Liu and coworkers (2016) evaluated the effect of 3D printed gelatin-alginate scaffolds in skin injury healing in mice. In this study, injury closing, and wound healing were enhanced with the scaffold’s application compared to the control. Wound healing was faster (around 2 days), showing decreased necrosis and hemorrhage, and formation of thinner crusts. In addition, the treatment with gelatin-alginate scaffolds promoted granulation tissue formation and angiogenesis; allowed good healing with less accumulation of keratinized substances and increased the strength and tensility of the injury [80].

Apart from the polymers, the combination of different techniques has been assayed to improve the mechanical strength and produce dressing materials with personalized layers. In the light of it, Palo and coworkers investigated a way to combine two different techniques to produce a bilayer carrier. These dosage forms were prepared by solvent casting (SC) combined with 3D printing (SSE) or electrospinning (called by the author’s SC/3D and SC/NF, respectively). SC/3D carriers were prepared with PVA and sodium alginate and were able to absorb liquid without cytotoxicity. Skin bioadhesion using simulated wound fluid demonstrated the decrease in the adhesiveness of SC/NF carriers compared to SC/3D carriers or solvent casting films, which impact the dermal use (non-adherent dressing is preferred). Additionally, SC/NF carriers were demonstrated to be the best physical structure for cell proliferation and adhesion. An additional inkjet 3D printing step was evaluated to explore the deposition of aqueous ink droplets on these carriers, aiming to use a drug solution for drug delivery in the future. However, the droplet distribution was uneven in SC/3D carriers, while a uniform distribution was obtained in SC/NF carriers. Even so, the use of inkjet printing can be an important alternative to provide specific doses of drugs, such as antibiotics, to facilitate wound healing or improve the treatment of other dermatological diseases [91].

As discussed before, 3D printing allows the development of the most diverse dressing structures and architectures. Among the wide range of wound dressing configuration and properties, asymmetric wound dressing has gained space in the research field lately. Of note, these materials have the outstanding ability to mimic dermal and epidermal layers of the skin. The external layer often provides a dense matrix with low porosity and small pores, exhibiting, also, a hydrophobic character. These characteristics act as protection against external factors, such as bacterial infection, mechanical stability and radiation [47,102]. On the other hand, the internal layer provides hydrophilic materials with loose structure, high porosity and large pores. This layer is involved with cell adhesion, migration and proliferation, as well as wound exudate absorption [47,102]. 3D printing can be an interesting alternative for the design and production of this asymmetric wound dressing due to its feasibility in the personalization and production of the most diverse complex structures.

In this context, Miguel and coworkers (2019) developed an asymmetric 3D printed construct for skin tissue engineering purposes. The external dense layer was composed of PCL and a silk sericin electrospun membrane, acting as a protective skin barrier, whereas the internal layer was composed of 3D printed alginate and chitosan hydrogel to provide an ideal environment for cell proliferation and migration. The top layer exhibited excellent mechanical properties, similar to human skin, and the bottom layer had adequate wettability, porosity and cell supporting properties [90]. Wang et al. (2019) have also explored this kind of bilayered printed form. They developed a bilayer scaffold with PLGA and alginate hydrogel for deep wound healing. The structure mimicked the skin structure, since the top layer (PLGA) and the bottom layer (alginate), simulated the epidermis and dermis, respectively. This pharmaceutical form was printed by two different techniques: (1) electrohydrodynamic jet (EHD) printing for the PLGA layer, followed by (2) SSE, for alginate hydrogel layer, which was crosslinked with calcium ions to prevent degradation. The use of PLGA blocked *S. aureus* penetration into the structure, like the epidermis’ role. Alginate promoted cell adhesion and proliferation as long as the wound environment was moist, a condition guaranteed by PLGA layer protection. The scaffold accelerated the wound healing process, which was completed by day 12, promoting angiogenesis and deposition of collagen I and III [94].

Other algae-derived biomaterials are being explored in 3D printing and wound healing. Xylorhamno-uronic acid, extracted from ulvacean macroalgae, was modified by methacrylation and further photo-crosslinked to produce xylorhamno-uronic acid hydrogels. The hydrogel was successfully printed by SSE with the controlled fabrication of 3D structures. The 3D printed form showed excellent cytocompatibility with human dermal fibroblasts, making this novel 3D printed scaffold a promising alternative for wound healing applications [88].

#### 5.1.2. Nanocellulose

Nanomaterials derived from cellulose are known as nanocellulose and can be categorized into cellulose nanocrystals, cellulose nanofibers and bacterial nanocellulose. These materials have advantages, such as biodegradability, renewability, biocompatibility, and mechanical strength [103].

During the SSE technique, due to the limited mechanical properties of hydrogel inks, sometimes it is not possible to print multiple layers, as the structure collapses. Thus, cellulose nanofibrils can be an interesting strategy to improve the printability of hydrogels, since they present a shear-thinning and rapid consolidation behavior [89]. Moreover, cellulose nanofibrils are a promising material for wound dressing, as they have excellent characteristics, including bacterial growth inhibition, high water absorption, adequate mechanical properties and can form translucent structures [89,104]. In this context, different pre-treatments of nanocelluloses (2,2,6,6-tetramethylpyperidine-1-oxyl-TEMPO nanocellulose and C-Periodate nanocellulose) were evaluated in terms of 3D printability and antibacterial effect for wound dressing materials. Overall, C-Periodate nanocellulose ink formed a more solid structure, and TEMPO nanocellulose tended to collapse, which could be attributed to its low consistency. Besides, these materials inhibited bacterial growth, revealing a promising property for dressing materials [79]. More recently, Chinga-Carrasco et al. (2019) explored Sugarcane Bagasse source of cellulose nanofibrils and confirmed its non-cytotoxic potential. Cellulose nanofibrils ink presented an adequate shear thinning behavior and were successfully 3D printed by SSE to form an object with a grid design. The authors also combined the cellulose nanofibrils with alginate and cross-linked them with Ca^2+^. However, the alginate addition to the ink had its print fidelity reduced. Even so, the cross-linking reduced the 3D printed grid area [87].

Similarly, Espinosa and coworkers (2019) explored 3D printed hydrogels with porous structures containing TEMPO cellulose nanofibrils and alginate for wound healing purposes. SSE technique was successfully applied, and the hydrogels exhibited important water absorption capability, demonstrating the suitability of these materials for wound dressing materials [89]. Besides the aforementioned studies, Chekni et al. (2020) reported a sensing nanocolloidal hydrogel with an antibacterial effect using cellulose nanocrystals and carbon dots. The dual functionality of the hydrogel dressing is based on the ability to absorb Fe^3+^ by the nanofibrillar network of the hydrogel, depriving the bacteria of this ion, and thus inhibiting its growth. Once the Fe^3+^ is absorbed on the surface of the carbon dots, its photoluminescence is quenched, promoting a sensing capability. Hydrogel patches were successfully and readily 3D printed by SSE on a 3M Tegaderm film and could be used to cover the wound, allowing a sensing capability of Fe^3+^ absorption, without the need for unnecessary changes of dressing that can lead to pain and can impact the healing outcome [95].

#### 5.1.3. Chitosan

Based on its hemostatic and antimicrobial properties, chitosan has also been a highly explored polymer for wound dressing applications, being already available in the market, as HemCon™ bandage [105]. Similarly, chitosan has been widely used in pharmaceutical 3D printing with the most diverse purposes [106], including wound dressing. Intini et al. (2018) explored 3D printed chitosan-based scaffolds by an innovative extrusion-based 3D printing technique for the treatment of diabetes wounds in a rat model. The scaffolds showed good biocompatibility, cytocompatibility and no cytotoxicity against human fibroblast cells and keratinocytes, which were able to attach and colonize the printed form. In vivo tests suggested that these 3D printed forms were as effective in wound healing as the conventional available alternatives. However, no sign of infection was observed in the 3D printed chitosan scaffold group, while the group treated with the commercial one showed infection in the wound [84]. More recently, a blend of chitosan and bioactive glass were proposed to fabricate porous dressing structures to accelerate wound healing. Cryogenic extrusion-based 3D printing was used to rapidly cool the solution below the freezing point. Different bioactive glass concentrations were evaluated, and they had no influence on morphology and water absorption. However, above a concentration of 20%, there was a decrease in the tensile strength and elongation rate. In addition, bioactive glass concentration has impacted the antibacterial effect, cell proliferation and migration, as higher concentration led to better performance. In vivo studies in rats demonstrated that the highest bioactive glass concentration (30%) exhibited better wound closure and collagen deposition, compared to the other groups (positive group, negative group and scaffold containing 20% of bioactive glass) [101].

#### 5.1.4. Synthetic Polymers

Synthetic polymers, compared to natural polymers, have the advantages of homogeneous physiochemical properties and tend to be more chemically and mechanically stable and possess lower cost, which are important points in 3D printing [107]. Synthetic polymers are also employed together with natural polymers, as shown in Section 5.1.1 by Milajovic and coworkers and discussed in Section 5.1.1, with the aim to modify the hydrophobicity and biodegradability of the structures.

Nun and coworkers (2020) combined two different techniques to prepare wound dressings, comparing extrusion-based 3D printed and electrospun dressing. Four synthesized polyesters were blended with PCL with the aim to investigate the influence of polymer composition and thread size in wound healing outcomes. Compared to the control (no dressing), neither the thread size nor the polymer composition affected the normal healing outcome. On the other hand, all dressings improved wound closure compared to polymeric materials commonly used in the literature. Thread size affects angiogenesis and epidermal thickness. Compared to the 3D printed and the control groups, groups treated with electrospun dressing showed an increase in epidermis thickness. On the other hand, 3D printed dressing increased angiogenesis, probably as a result of their larger pore size [96].

The possibility of polymer synthesis based on the desired properties is another great advantage of synthetic polymers. Based on that, Streifel and coworkers (2018) proposed hydrogel foams of synthetized PEGMA-sodium acrylate-PEGDA copolymer, since these gels are extremely absorbent, and further incorporated with kaolin, a hemostatic aluminosilicate. There are products on the market, such as QuikClot Combat Gauze (QCCG, Z-Medica, Wallingford, Connecticut, EUA, composed of kaolin, which is able to address hemostatic activity. However, this product suffers from low fluid absorption potential and may be difficult to remove from the skin. The proposed gels were produced by the high internal phase emulsion technique and showed good viscosity and shear thinning behavior for 3D printing. The hydrogels showed cytocompatibility with human dermal fibroblasts, high fluid absorption and good hemostatic activity, revealing the feasibility of this multifunctional material to prevent several hemorrhages. Furthermore, these 3D printed gels have demonstrated high fidelity to the design and hierarchical porosity [85].

3D printing can also be used to create a dressing with no intrinsic activity attributed, but to aid as a physical top layer. Chuan and coworkers (2018) reported the printability of ABS curved skin, fitting the exact area of the human wound. The approach comprised the use of 3D printed ABS artificial skin and the addition of a hydrocolloid gel composed of agar-agar and crushed eggshells. This gel composed the inner layer of the artificial skin to provide a natural adhesiveness, as well as keep the wound cool. Data demonstrated a short drying time with good adherence to human skin for a long time. Besides, after the 3D printed skin was peeled off, no adhesive marks or stains were observed, which could be of great value for burn wounds [83].

#### 5.1.5. Other Polymers

However, despite all these results, one important concern in wound healing treatment is the material ability on minimizing the complex microenvironment of the natural extracellular matrix (ECM) [108,109]. Commonly available polymers, natural or synthetic, lack on this task. To overcome this issue, decellularized extracellular matrices are becoming promising materials, as they maintain the structural proteins of ECM, as glycoproteins, glycosaminoglycans and biologically active networks [108]. However, decellularized matrices have some disadvantages since membranes or gels of these materials have limitations to form three-dimensional porous structures. In this sense, 3D printing at low temperatures becomes an approach to circumvent the problem. Based on that, scaffolds composed of decellularized small intestinal submucosa were 3D printed by novel cryogenic bioprinting assisted by free-form extrusion and were proposed as a new strategy for skin tissue engineering applications. Addressed by the limitation of 3D printing of decellularized materials, the novel printing technology developed by the authors consists of a 3D robot platform, a pneumatic dispensing system and a cryogenic stage. The scaffolds were successfully printed and demonstrated great mechanical properties, allowing the maintenance of the porous structure, which could support cell growth. Even so, pore size had impacted the mechanical properties once the increase of pore size led to a decrease of young’s modulus. In vitro experiments demonstrated that the 3D printed scaffold could accelerate the production of ECM proteins and also cell proliferation [92].

In the current scenario, the data discussed here evoke great possibilities of 3D printing in skin injuries and wound healing. Interactive dressing can be developed by 3D printing with the manipulation of the most diverse materials, providing multifunctional properties to accelerate the healing process. There is a trend in the use of polymers and materials that have intrinsic activities, such as antibacterial activity, fluid absorption capacity, hemostatic activity, among others. This can provide several advantages for the development of interactive dressings and can significantly impact the quality of life of patients. Still, 3D printing also allows the production of inert dressings, which can be easily associated with another pharmaceutical strategy.

In addition, it must be highlighted that some studies have put some effort into the development of dressings combining two distinct techniques, either two different 3D printing techniques on the same dressing, or different techniques, such as 3D printing combined with electrospinning. This interesting trend can give some new insights for future research.

Despite all the promising outcomes, 3D printing has limitations regarding the printing technique and the polymers used, as limited printability and low mechanical properties. Here several approaches to improve the mechanical properties of these dressings were discussed, which cover the use of polymeric blends, the addition of nanofibers, extrusion at freezing temperatures and hybrid 3D printing techniques.

Even though there are still few studies on the development of plain dressing by 3D printing, this technology holds a promising future regarding the feasibility of complex structures, a wide range of polymers and personalization of wound dressing and skin substitutes. On the other hand, progress in the optimization of printing properties to improve the performance of the materials and to produce customized therapeutic skin dressing has been observed in the last few years, which highlights the potential application of 3D printing and the perspectives of further studies in this area.

### 5.2. Active Ingredients Delivery

Cutaneous topical treatment with active ingredients may be required by some skin conditions, as injuries, inflammation, or infections, among others. Some factors can compromise patient adherence to topically applied treatments, including complex treatment regimes, misunderstanding of side effects and disease, drug affordability, non-aesthetic approaches, and formulation properties (smell, spreadability, bad organoleptic properties, discoloration and messiness of the delivery system during use). Dose inaccuracy can also be a problem, as conventional formulations do not allow precise control of drug dose and release. Therefore, maintaining treatment adherence can become a challenge for both, the patient and the physician, and compared to oral administration, may lead to more time-consuming and complicated treatments [110].

Other concerns about topical skin delivery are allergic reactions and the high frequency of dose applications, which could be overcome through innovative technologies [28]. Based on this scenario, new topical cutaneous drug delivery systems that can provide more precise doses and drug release rates, targeting specific skin areas, and be focused on the patient’s needs, would be very much appreciated.

3D printing allows the production of complex formulations containing active ingredients for the development of new pharmaceutical dosage forms, considering tailor-made-dosage forms for individual differences. This new technology can produce customized shapes, size, porosity, and a number of layers of dressing/mats/devices while allowing a precise personalization of active ingredients dose and release profiles [111,112].

Active ingredients can be incorporated in 3D printed products by different approaches, which depend on the 3D technique, the materials and the properties of the active ingredients: pre-printing (e.g., blended with polymers prior the filament production by HME [113] or together with the polymers ink prior SSE [9] or SLA [113]) or post-printing (e.g., soaking the 3D structure on a drug solution or suspension [70]). In any case, as will be discussed in the following sections, an active ingredient can be also placed outside the 3D printed product and/or can be infused or injected into the structure when desired.

The incorporation method can also provide different drug release profiles and drug loading values, which can be used as a strategy to modulate drug delivery [114,115]. Most of the studies discussed here incorporated the active ingredient in the materials before the 3D printing process.

Table 2 summarizes the studies concerning 3D printed products for active ingredient delivery across the skin. In the following sections, we will discuss different methods and 3D printing applications for topically and localized active ingredient delivery to the skin, focusing on its release properties. We will present the active ingredients following the sequence: drugs; peptides and proteins; metals; and natural compounds.

#### 5.2.1. Drugs

Several skin pathologies imply the use of pharmacological drugs for efficient treatments [155]. Acne [156], psoriasis [157], skin infections [158] are some examples that often require topically applied and skin localized drug delivery. A variety of drugs are intended for topical use against these conditions, such as antibiotics and antifungals, for skin infections; topical corticosteroids, for skin inflammations; topical retinoids, for acne treatment; and local anesthetics, as lidocaine, for dermal anesthesia. A great range of skin diseases can be treated with topically applied drugs, thereby avoiding the systemic side effects [35]. These drug classes have been further evaluated to obtain versatile and personalized delivery dosage forms by using additive manufacturing.

Maver and coworkers (2018) developed a pain-relieving wound-dressing combining a 3D printing technique and electrospinning. Diclofenac sodium and lidocaine, a nonsteroidal anti-inflammatory and a local anesthetic, respectively, were loaded in dressings produced by SSE with alginate and carboxymethylcellulose. The drugs were added before the 3D printing process, what did not significantly affect the printability of the hydrogels. Lidocaine and diclofenac sodium showed a sustained release from the 3D printed hydrogels over a period of 48 h. Approximately 25–30% of the drugs were released immediately, followed by a fast release until reaching about 75–80% (350 min), and by a further slower release phase in the next hours (up to 48 h). Besides, the release profile was similar for both drugs, indicating that it was not affected by the drug’s chemical composition. Electrospun drug-loaded fibers exhibited a faster drug release, as 93% of diclofenac and 78% of lidocaine were released within the first 30 min. Therefore, the authors proposed combining both techniques in one wound dressing. The first layer was composed of lidocaine-loaded electrospun fibers, which would trigger immediate pain relief, and the second layer was composed of diclofenac 3D-printed hydrogel, continuing pain relief for 2 days [9].

One year later, Long and coworkers (2019) designed a 3D printed chitosan-pectin hydrogel loaded with lidocaine for wound dressing. Chitosan has important advantages for skin applications, as it is bioadhesive, biocompatible, biodegradable and non-toxic, and shows antimicrobial, antioxidant, hemocompatible and hemostatic activity [20,159]. The authors prepared hydrogel scaffolds by SSE followed by lyophilization. To provide the best wound environment, the scaffolds showed a high swelling ratio and water absorption, highlighting their ability to absorb exudates and retain wound moisture. Bioadhesion strength indicated great self-adhesion onto the skin. Wound dressing should be self-adhesive but easy and painless to remove. Different drug concentrations were evaluated, and all formulations showed a fast drug release within the first hour, followed by a sustained release behavior. This release profile was attributed to the lidocaine entrapment within the polymers [123].

Chitosan films were also proposed by Hafezi and coworkers (2019), using the jet-dispenser 3D printing technique. Fluorescein sodium was used as a model drug, along with genipin, as a crosslinker molecule with anti-inflammatory and antibacterial activity, and PEG and glycerol, as plasticizers. In vitro mucoadhesion studies showed their ability to adhere to a model mucosal surface, in which the plasticizer could play an important role, as films containing PEG hydrated better, leading to a higher detachment force. The high adhesion reinforced the potential of these chitosan films to stay longer when applied to the skin wound surface, therefore, contributing to better patient compliance. For the release studies, fluorescein sodium was used as a model drug. Approximately 67% of fluorescein sodium was released within the first hour, which was in agreement with the swelling study, which showed fast hydration in the first 15 min [121].

The use of protein-based biomaterials has been vastly spreading in biomedicine, tissue engineering [160] and drug delivery [161] and becoming an important natural alternative to synthetic polymers. These materials are interesting in drug delivery because they have low toxicity, are water-holding, able to emulsify and are abundant in nature [162]. Besides, protein structures can enhance cell attachment and migration [163]. All these features sustain their use on topical dressings for skin diseases, such as burn wounds.

Navarro and coworkers (2020) successfully developed a keratin scaffold loaded with halofuginone for burn wound healing treatment. Halofuginone is an FDA-approved inhibitor of collagen I synthesis and prevents the accumulation of abnormal fibrillar collagen, which is associated with fibrosis and wound contracture. The DLP technique was used to fabricate keratin-based scaffolds, followed by lyophilization and gamma irradiation sterilization. Noteworthy, the results showed that lyophilization, sterilization and further rehydration prior to in vivo use, can lead to changes in the crosslinked network, in the dimensions of the samples and/or loss of mass. In vivo, the porcine burn wound model showed that 3D printed halofuginone-loaded scaffolds were non-inferior to the treatments in the literature, and do not hamper the healing processes. However, the 3D printed scaffolds were the only group to significantly demonstrate improvement in healing from day 30 to day 70, which could be an indicator of more organized dermal healing after-burn. As concluded by the authors, this treatment is slower, however, improves healing [135].

Wound infections can be an important issue during the wound healing process, as they may delay the healing progress and impact negatively on pain and life quality [164]. Singh et al. (2021) designed and characterized FDM 3D printed neomycin PLA mats for wound covering and antibiotic delivery. The PLA mats were printed and soaked in molten PEG or in molten PEG containing neomycin for the drug load mats. Three different molecular weights of PEG were evaluated (400Da, 6kDa and 20kDa). Results showed that the higher the molecular weight of PEG, the lower the total drug cumulative release over a 3-week period. Additionally, coating with PEG 400 Da exhibited great mechanical properties, evidenced by the formation of a flexible mat, with sufficient porosity, making it suitable for dermal/bandage-like wound covering [152].

In the same context, mupirocin, a topical antibiotic, loaded in 3D printed scaffolds were proposed to cover piercing studs and prevent infection. The bioabsorbable PLGA “biopierce” was designed to stay in the human tissue for up to two weeks and release the drug during this period to prevent infection and allow wound healing. The antibacterial assay demonstrated bacterial inhibition over 14 days, showing the efficacy of the 3D printed product. This study brings new insight into drug-eluting wound dressings [134].

Stimulus-responsive systems for the delivery of therapeutic drugs are another interesting approach for topical treatments. In this context, Niziol et al. (2021), proposed a thermoresponsive hydrogel loaded with Octenisept^®^, an antimicrobial agent composed of octenidine dihydrochloride and 2-phenoxyethanol, for wound healing applications. SSE was employed for the fabrication of these hydrogel-based dressings composed of poly(N- isopropylacrylamide) (PNIPAAm) precursors, sodium alginate and methylcellulose. The swelling hydrogel and drug release profiles were dependent on the temperature. The highest the temperature, the lowest the swelling ratio and the highest the OCT release [148].

Another research group has developed single-step EHD printing for rapid tailored dosage forms containing antibiotics for wound healing. Amoxicillin, ampicillin, and kanamycin were individually mixed with bacterial cellulose and PCL to fabricate 3D printing patches. The complete drug release took 14 days, with an initial burst release, followed by a slow-release phase. Interestingly, the kinetic releases fit Fick’s law of diffusion, regardless of the solubility of the antibiotic. In addition, the antibacterial assay demonstrated that all patches were effective against gram-positive and gram-negative bacteria. This study provides a versatile approach for the development of wound dressing with antimicrobial drugs [138].

To investigate the influence of 3D printed parameters on fiber deposition and alignment and to evaluate the influence of the composition of the patch material and the print-void geometry on drug release, Wang and coworkers (2017) developed 3D printed film patches via EHD technique. Films were composed of PCL or PCL-PVP loaded with an antibiotic (tetracycline). Drug release from PCL patches was slower than from PCL-PVP patches, probably due to the higher hydrophobic property of PCL. In addition, the pore size had an important influence on tetracycline release, in which patches with lower pore sizes values (200 × 200 μm^2^) showed a relatively slower release (65.4 ± 1.6%) compared to patches with higher pore sizes of 500 × 500 μm^2^ (72.1 ± 2.9%), in the first 24 h. For all samples, after this burst release, a slow and steady release was observed until the fifth day. The authors showed that both matrix composition and pore size can be tailored, based on the aimed drug release profile, providing appropriate drug concentration and release to inhibit bacterial infection [117].

Tetracycline was also used by Dominguez-Robles and coworkers (2019), in addition to a model compound, curcumin. Lignin, a natural biopolymer with antioxidant properties, blended with PLA and castor oil, was used to prepare 3D printed filaments by HME. Tetracycline was incorporated with lignin in the filament and the FDM technique was applied to produce the 3D-printed meshes. Even so, due to the printer configuration, a two-nozzle FDM was used to combine the mesh with a printed PVA film (as the second layer). Finally, a curcumin film, produced with hydroxypropyl methylcellulose (HPMC), was placed on the top of the 3D-printed mesh. The mesh dimensions affected the curcumin release rate, and so the meshes might be designed according to the patient’s healing needs [120].

Despite many studies on this area aimed at wound healing applications, there are still many other skin diseases and possibilities to explore by using 3D printing techniques in the development of therapeutic approaches for skin delivery. Bom and coworkers (2020) developed a drug delivery system for topical application, based on alginate-pregelatinized starch. Semisolid extrusion was chosen as the 3D printed technique, and the process parameters were evaluated by a Quality by Design approach. The addition of starch led to an increase in the size and the number of open pores, which can influence the drug release. In the in vitro rhodamine B release, alginate-starch patches demonstrated a higher burst effect than alginate alone, which was explained by the starch swelling properties. After 360 min, alginate-starch patches released 90.46% vs. 71.51% of the alginate patches. According to the authors, these data pave the way for future drug delivery strategies for topical skin delivery [111].

Moreover, the alliance of 3D printing and nanotechnology has been widely explored in the biomedical area in the last years and can improve pharmaceutical dosage forms [8]. Nanotechnology plays a prominent role in developing more complex drug release profiles, and in targeting specific pharmacological therapy [165]. Wang et al. (2020) explored 3D printed hydrogel loaded with cryptotanshinone (CPT) niosomes for topical therapy of acne. The 3D printed hydrogels (blank hydrogel, CPT loaded hydrogel and CPT niosomes loaded hydrogel) demonstrated good bioadhesion, gel strength and extrusion. Furthermore, in vitro release showed that CPT niosomes loaded hydrogel were able to control drug release, even up to 96 h. It exhibited a lower release (61.5%) compared to the CPT-loaded hydrogel (78.6%). Skin permeation and skin deposition experiments demonstrated, respectively, significantly higher rates of transdermal flux and skin deposition of CPT from 3D printed niosomes-loaded hydrogels. These results highlighted the feasibility of 3D printing to incorporate nanostructures (niosomes) for enhanced topical drug delivery. Moreover, the in vivo anti-acne assay was promising, showing an anti-acne effect without skin irritation and holding great potential for novel personalized acne treatments [136]. However, as highlighted by Santos and coworkers (2021), the association of nanotechnology and 3D printing for topical use must be carefully evaluated to avoid toxicity and systemic absorption of the drugs [8].

Among the most important advantages of using 3D printing in the pharmaceutical field is the possibility of personalization, especially for topical products where it is possible to design the product, based on the patient’s needs. Goyannes and coworkers (2016) explored the potential of 3D printing to produce personalized nose-shape anti-acne devices loaded with salicylic acid by two different 3D printing techniques: FDM and SLA. Salicylic acid was blended with the FDM polymers (Flex EcoPLA—FPLA—and PCL) and the filaments were produced by hot-melt extrusion. Thermal degradation of the drug was observed during the extrusion because of the high temperatures used. An FPLA-salicylic acid filament was efficiently printed as a nose-shape mask instead of a PCL-salicylic acid filament, which was not possible to print. For the SLA printing, the drug was mixed with both polymers (PEGDA and PEG) before solidification by the laser beam. There was no drug degradation, and the printing resolution was better than by FDM technique. Drug diffusion from 3D printed circular-shaped devices produced by SLA exhibited a faster release profile than those prepared by FDM. According to the authors, SLA printing is a promising technology to produce personalized anti-acne devices and could allow specific dosage, shape, and size [113].

From the data discussed above, 3D printing has demonstrated outstanding positive points in producing complex and customized drug delivery systems for topical skin delivery. The burgeoning area is an important technology that can provide better therapeutic results for skin diseases, although it still needs to be explored more. There is still a gap in the evaluation of drug permeation and penetration across the skin. Moreover, considering the site-specific/local drug action, it is extremely important to ensure that no systemic absorption of the drug will occur by the application of the proposed 3D printed products.

#### 5.2.2. Peptides and Proteins

Topically applied proteins and peptides for the treatment of skin disorders have also become an important research field in the last years [166]. These macromolecules are relevant for the local treatment of several skin diseases, and increasing their site-specific delivery is an important goal to be achieved [26]. However, peptides and protein delivery through the skin suffer from inefficient penetration and permeation. This limitation results in reduced efficacy and wastage, caused by the inadequate/insufficient drug concentration at the site of action [133]. Some approaches have been proposed to improve their skin delivery, such as the use of microneedles (MN).

MN are a very explored alternative for the delivery of these molecules and are normally referred to as transdermal systems, but they can also represent a strategy for localized site-specific drug delivery [26,167]. The fine control of the microneedle length and arrays allow the targeting of ingredient actives to the epidermis or the dermis, producing minimal systemic effects [26,133]. The use of 3D printing to produce MN enables their customization, such as distribution and depth of needles and also the drug release rate [18]. Remarkably, this technology provides continuous one-step manufacturing and cost-efficiency [18,52]. Its use to improve the skin delivery of peptides and proteins for local treatment will be discussed in this section.

Acetyl-hexapeptide-3 (AHP-3) is a small topically applied anti-wrinkle peptide, with good efficacy and safety characteristics, but its permeation across the skin is limited due to its high molecular weight and hydrophilicity. Lim et al. (2020) developed a 3D printed personalized microneedle patch as a “poke and patch” approach for AHP-3 solution delivery. Although the authors referred to transdermal delivery, this strategy was proposed to promote the local effect of AHP-3. DLP technology was used to fabricate MN with different geometries and curvatures, and later to fabricate a personalized MN patch for the periorbital eye region. 3DM-castable resin was used as the main material. MN geometries with different curvatures were produced in order to analyze the relationship between different MN dimensions and their effect on mechanical strength and skin penetration efficiency. The author’s hypothesis was that each curvature would have its own optimized MN geometry. However, the results showed the same optimized geometry for all three curvatures (MN height of 800 μm; tip diameter of 100 μm, interspacing of 800 μm and base diameter of 400 μm). In vitro drug delivery demonstrated that the optimized personalized curved MN patch for the eye region provided approximately 45x higher AHP-3 delivery through the skin, compared to a flexible commercial MN patch [133].

Peptides can also be loaded within the needles, and so, the same authors proposed an MN patch loaded with AHP-3 [145]. Due to the few available photocurable resins for drug loading and delivery uses, PEGDA and vinyl pyrrolidone (VP) were applied in the fabrication, by DLP, of microneedle patches. Different ratios of PEGDA and VP were evaluated and demonstrated no degradation of AHP-3 during the process. An optimal resin ratio was chosen for the fabrication of a 3D scanned face personalized patch. MN had good skin penetration and mechanical properties. Besides, the personalized patch provided standardized dosing, providing a single-step procedure for the patient. Therefore, these MN are an interesting approach for anti-wrinkle therapy and could be translated to the delivery of other peptides [145].

Topically applied drugs can suffer from bad penetration if a wound crust exists, being miniaturized needles a great option. In the light of personalized dressings, a very innovative individualized miniaturized needles bandage with wireless controlled drug delivery for diabetic chronic wounds was proposed by Derakhshandeh et al. (2020). The FDM technique was used to fabricate hollow miniaturized needles that could deliver vascular endothelial growth factor (VEGF) into deeper layers of the wound bed. A drug solution was infused into the miniaturized needles by a wireless stimulus. MN were tested using an in vitro method, which mimics the necrotic tissue and a crust covering the wounds. Due to the increased bioavailability of VEGF below the wound crust, the data demonstrated faster closure rates for miniaturized needles than the topically applied VEGF. In vivo studies also showed a better healing performance for the miniaturized needles compared with the topically applied VEGF. This versatile platform was proposed to deliver multiple drugs, modulate the drug release and for medical administration through in-house smartphone commands [130].

The same research group compared VEGF delivery from 3D printed hollow miniaturized needles, liquid jet injectors and VEGF topical application, which promoted different drug spatial distribution over the wound bed. 3D printing of hollow needles was conducted by the FDM technique, with a Luer Hub design, which could be fixed on any standard syringe for the drug infusion and delivery. In vivo studies showed that VEGF delivery by miniaturized needles improved quality and healing rate, compared to the topically applied group. These effects were attributed to the distribution profile of VEGF through the miniaturized needles, affording a more uniform drug distribution through the wound area, and also a more accurate delivery to the target point, compared to the other methods [149].

Stimuli-responsive delivery systems are smart approaches for the controlled and site-specific release of molecules [168]. Siebert et al. (2021) proposed a multifunctional wound hydrogel patch containing VEGF decorated with photoactive tetrapodal zinc oxide and activated through ultraviolet/visible light stimulus. The stimulus-responsive system provided controlled release of VEGF with green light exposure over 120 s. Two concentrations of VEGF were studied and the release rate was not affected by VEGF concentration. Additionally, tetrapodal zinc oxide features antimicrobial activity, which is important to the adequate wound healing process, in agreement with the in vitro antibacterial assays. The in vivo studies of the wound healing model showed that the 3D-printed scaffold with tetrapodal zinc oxide and VEGF promoted faster wound healing, by enhanced anti-inflammatory, angiogenesis and cell proliferation effects [151].

Diabetic wound healing is still a big challenge for physicians and formulators, mainly because of the sustained chronic inflammation. Wan et al. (2019) developed a bilayer skin substitute, with a top layer composed of silver-loaded gelatin cryogel and a bottom layer with platelet-derived growth factor-BB (PDGF-BB). The silver top layer was designed to protect the skin against bacterial infection (see Section 5.2.3), as it is an effective antimicrobial metal. PDGF-BB is a growth factor, widely used in diabetic wound healing. PDGF-BB was loaded with semisolid extrusion ink, whereas silver nanoparticles (AgNP) were incorporated by impregnating the top layer in a GelMA nanoparticle-loaded solution. In vitro drug release of PDGF-BB had an initial burst release followed by a sustained release over 14 days. However, after 14 days, AgNP release was still not completed. In vivo studies showed that the scaffold was able to provide collagen deposition, new blood vessel formation, re-epithelialization and the formation of granulation tissue [125].

Melt-electrowriting technique was applied to create scaffolds loaded with milk proteins for skin regeneration purposes. Before the 3D printing process, PCL was blended either with whey protein or lactoferrin, or both (COMB). 3D printed scaffolds showed high porosity and low degradability. Qubit^®^ Protein Assay Kit was applied to evaluate the total protein release, showing fast protein release from the scaffold within the first hour (40% approximately). A gradual increase in the release was observed till day 21, reaching final percentages of 50.6%, 67.9% and 78% of total protein released from the scaffolds containing lactoferrin, whey protein and COMB, respectively. Interestingly, scaffolds loaded with both proteins had a significantly higher total protein release than scaffolds with lactoferrin and whey protein alone. Noteworthy, in vitro models with human keratinocytes and normal human dermal fibroblasts cells demonstrated significantly enhanced cell infiltration, spreading and growth for scaffolds with lactoferrin and with both proteins compared to scaffolds composed only by PCL [122].

The delivery of proteins and peptides by the topical route is still a challenge. 3D printing can be suggested as an interesting alternative to produce skin delivery devices for proteins and peptides, highlighting the benefits and versatility of this technology. Different approaches have been reported, varying from microneedles to protein-loaded scaffolds, which can improve the delivery of active ingredients and, also in vivo wound healing outcomes.

The use of proteins and peptides presents some pharmacotechnical challenges, such as protein instability, and biological challenges, as they can trigger important immune responses [166]. Still, as it is a very recent area and studies involving 3D printing are in a very early stage, limitations of instability and immunogenicity have been little explored in studies involving this technology. However, we hope these issues will be further explored and evaluated, as future investigations are important to ensure their translation to the clinic. 3D printing can be a great ally to overcome the challenges of topical delivery of peptides and proteins and still provide personalized treatment for each patient.

#### 5.2.3. Metals

Metal compounds have an important role in wound healing and scar formation, since they are catalysts of important enzymes, structural elements of proteins and transcription factors, and can also modulate their activity through conformational changes. Metals can regulate different phases of the healing process: hemostasis, inflammation, proliferation or maturation, and their use along with traditional medicine can improve these processes [46].

Regarding the metal’s properties, zinc is crucial in various physiologic processes, such as growth, immune function and wound healing. It has also been suggested that this metal disrupts bacterial cell membranes and stimulates skin regeneration by cell proliferation [46,169]. Copper can affect the membrane integrity and vital functions of microorganism’s cells and promotes angiogenesis in the wound area, via induction of vascular endothelial growth factor (VEGF) [46,170]. Silver causes the lysis of bacterial cells and can modify vital functions like energy production, although there is still a concern about its toxicity [171]. Manganese, an important metal, when bound to the enzyme superoxide dismutase can affect collagen contraction. It is also associated with protection against UV-induced photoaging and with accelerating wound healing [46,172] Potassium permanganate (KMnO4), a strong oxidase agent, is widely used for the treatment of wounds, disinfections and for inflammatory skin diseases, such as contact dermatitis and psoriasis [173,174].

Metals could play an important role in wound dressing, as they are able to interfere in antimicrobial activity and, also, positively influence the healing process. This section summarizes studies that use metals as active components associated with 3D printing in the development of new wound dressings.

Muwaffak et al. (2017) developed 3D printed antimicrobial wound dressings loaded with zinc, copper, and silver ions. The filament was produced by hot-melt extrusion using PCL, and metal ions were incorporated within the polymer, followed by FDM printing. Stronger dressings were achieved with the increase of shells, although this increase led to less quality of the structure and longer printing time. Silver release in the first 24h was very fast but it slowed down in the subsequent hours. Zinc and Copper release followed the same trend, but with a slower burst release phase. PCL acted as a barrier to the release of the metal due to the slow water penetration in its polymeric matrix. As suggested by the authors, controlled release is an interesting approach to inhibit bacterial growth in the wound environment since the metals could be sustainably released during the whole treatment. To evaluate the possibility of printing specific dressing for each patient’s anatomy, a nose and ear shape dressing were successfully printed based on the 3D scan model, evoking great possibilities in the personalization of shape and size [112]. Azadmanesh et al. (2021) also used copper, but combined with carbon dots, to develop 3D printed scaffolds for wound healing applications. Carbon dots are carbon nanomaterials that hold great advantages as antimicrobials for biomedical products, as they possess a high surface-to-volume ratio and can be modified with metals to increase their antibacterial effect. The printed forms were produced by FDM using PLA, and the core of the scaffold was comprised of a mixture containing copper carbon ducts (Cu-CD), rosmarinic acid, hyaluronic acid, and chitosan hydrogel, by adding the mentioned mixture into the 3D printed scaffolds. The scaffold showed antibacterial activity, no cytotoxicity and good in vivo wound healing results, which was confirmed by gene expression of PDGF, TGF-β, and MMP-1 and histological analysis [139]. Hyaluronic acid was used in an innovative in situ printing system proposed by Hakimi and coworkers (2018). Although the article is focused on cell printing, they demonstrated the in situ printing of an acellular homogeneous hemostatic barrier composed of fibrin and hyaluronic acid in a wound healing porcine and murine model, illustrating the ability to deposit the material on surfaces with respiratory movement [175].

As previously discussed, silver ions have a great potential in preventing microbial growth in the wound. Afghah and coworkers developed 3D printed scaffolds based on polycaprolactone-block-poly(1,3-propylene succinate) (PCL-PPSu) copolymers loaded with silver nitrate, which was incorporated by polymer impregnation before extrusion. In this study, copolymer PCL-PPSu was used to improve the physical and mechanical properties of the filaments, with lower processing temperature compared to neat PCL. This is of great value for the incorporation of thermolabile actives. The 3D printed scaffold showed higher hydrolytic and enzymatic degradation behavior and improved hydrophobia. The scaffold was designed to have a porous structure, with interconnected pores, to keep good oxygenation of the wound environment. Silver release was slow (about 4.5–7.5 mg kg^−1^) and below cytotoxic levels. PCL and PCL-PPSu loaded scaffolds exhibited antimicrobial activity against the microorganisms tested: *C. albicans* > *E. coli* > *S. aureus* > *P. aeruginosa* [127]. All these properties and characteristics made these scaffolds suitable for wound dressing.

Among the inorganic nanomaterials, metallic nanoparticles have been widely used for wound healing purposes due to their antibacterial activity. These particles are characterized by small size and high surface area, which tend to impact cellular uptake [176,177]. Shi and coworkers (2019) explored the possibility of using a 3D printed dressing membrane loaded with AgNP and oil infusion for infected wound healing treatment. In this study, polydimethylsiloxane (PMDS) was used as the main polymer matrix for SSE printing, due to its biocompatibility and non-toxic properties. Silicone oil infusion was used to promote better PMDS non-fouling properties and a slippery surface. Silver release from the membranes was sustained, which is ideal for wound treatment. The dressings did not show cytotoxicity and its slippery surface repelled blood on its surface leading to no blood staining. Regarding antibacterial activity against *E. coli* and *S. aureus*, the printed form inhibited bacterial adhesion and growth [124].

Wan et al. (2019) used AgNP for the same purpose as described above. The printing ink for SSE was prepared with GelMA and PDGF-BB, and then they were impregnated in AgNP solution. AgNP release was not complete in 14 days and its release rate was much slower than PDGF-BB, which was almost completely released in 7 days. Considering the antibacterial effect, the bilayer scaffold loaded with PDGF-BB and AgNPs inhibited the growth of *E. coli*, *P. aeruginosa* and *S. aureus*, as detected by CFU counts [125]. Multifunctional 3D printed wound dressing was also proposed by Alizadehgiashi and coworkers (2021) combining small molecules (e.g., gentamicin), metal nanoparticles (e.g., AgNP) and proteins (e.g., VEGF). The hydrogel ink was composed of chitosan and cellulose nanocrystals and exhibited shear-thinning and fast viscosity recovery properties. The release profile of the active ingredient was independently controlled by the number of filaments in the dressing. Compared to controls, in vivo wound dressing studies containing AgNP and VEGF demonstrated improvement in granulation tissue formation and differential levels of vascular density, the latter being influenced by the type of growth factor release profile (extended or short release profile) [137].

3D printing can also be applied in the development of porous templates, as proposed by Wu and coworkers (2019). PLA porous templates were produced by FDM and hydrogel precursors were cast in the porous templates, with consecutive template removal. Thus, super porous polyacrylamide/HPMC hydrogel dressing containing AgNPs cross-linked in the hydrogel matrix was produced, demonstrating desirable cytocompatibility, antibacterial activity, rapid water uptake capacity, and in vivo, a faster wound healing rate when compared to other dressings. The interaction between AgNPs and ethylene groups of the hydrogel precursors provided an organometallic complex with controlled release of AgNPs, which positively impacted the balance between cytocompatibility and antibacterial activity [126].

The use of dressings with metals is already a reality in the market, but 3D printing brings the proposal of personalization of these dressings, which is important for treatment adherence and individualization of therapy. Metallic nanoparticles proved to be very prominent, which demonstrated to be viable for 3D printing of dressings, promoting more complex and multiscale structures. On the other hand, studies with 3D printing are still quite recent, which raises important points of stability, permeation and safety of these materials. In particular, metals can be harmful if absorbed through systemic circulation, and thus, careful analysis of their permeation parameters is of great interest. Mainly two printing techniques were used, FDM and SSE, leaving doors open for the insertion of metals in other 3D printing techniques. 3D printed forms loaded with metal particles are suitable for novel wound treatment and dressings, which can be further explored for the development of new medical approaches that can be a real possibility in the near future.

#### 5.2.4. Natural Compounds

Natural compounds have been reported as a promising alternative against infections. They have been traditionally widely explored for wound healing treatment in various cultures, mainly based on the use of plants as primitive wound dressings, and late by researchers, who confirmed the antibacterial, antioxidant and antifungal activity of certain compounds, among other activities [178,179].

Aranci et al. (2020) investigated the properties of 3D printed scaffolds loaded with propolis extract. Propolis is a type of resin synthesized by bees for hive protection, with well described antibacterial, antifungal, antiviral, anti-inflammatory, and antioxidant effects [180]. These activities are of great interest for wound healing applications. In the study, SSE was chosen as the printing technique and the main polymer was sodium alginate, which was mixed with propolis extract to prepare the printing ink. At a pH value of 7.4, cumulative propolis release from the 3D printed scaffolds reached close to 99% in 4h, being, therefore, suitable for controlled release. Even at a pH of 2.0, a drug release close to 97% was reached in a few hours, which is relevant, since the wound environment is usually acidic. Swelling tests were carried out to evaluate the water absorption ratio of the dressing, a crucial parameter to ensure the movement of body fluids inside the scaffold. Ideally, this ratio should be high enough but not lead to its fast disintegration. The test showed that the scaffolds loaded with propolis had lower porosity and absorbed less water than the blank ones, which is a positive outcome for wound healing. The scaffolds showed antibacterial activity against gram positive bacteria [129].

Another concern in this kind of treatment is the dehydration of the wound, which may result in a slower healing process. Andriotis and coworkers (2020) developed 3D printed patches made of natural-based materials: pectin, Manuka honey, and chitosan/β-ciclodextrin/propolis extract complex (CCP) prepared by SSE. Propolis was chosen as the active ingredient and the aim of their study was to use the patches to maintain the wound’s moisture and protect it from infections and contamination. Patches containing CCP showed antimicrobial activity against S. aureus. CCP concentration was also relevant to determine patch transparency, which is useful to evaluate the wound without changing the dressing. Patches containing < 5% CCP were transparent. In vitro wound healing tests, showed that CCP enhanced the healing properties of pectin/Manuka honey patches [128].

Aloe vera has also been widely studied in wound healing due to its outstanding anti-inflammatory, antioxidant, and antimicrobial effects [181]. In addition, some essential oils have gained space in the pharmaceutical field, e.g., eucalyptus essential oil, and have been investigated for wound healing purposes, based on its terpene’s analgesic, anti-inflammatory, antioxidant, antifungal, and antiradical activities [182]. Karavasili et al. (2020) produced 3D printed scaffolds loaded with three different natural compounds: Manuka honey, Aloe vera and eucalyptus essential oil. Alginate and methylcellulose were used as polymers and the active ingredients were mixed with the polymers before SSE 3D printing. Mechanical studies, by nanoindentation and finite element analysis, demonstrated that different concentrations of the bioactive components can influence the tensile strengths of the scaffolds, such as the elastic modules, which decrease with the increase of Manuka honey and aloe vera. All the printed scaffolds showed antibacterial and antibiofilm activity against gram positive and negative bacteria. The in vitro wound healing assay, based on a cell monolayer vertically scratched, demonstrated that all hydrogel scaffolds were able to induce full wound closure (after 48 h), compared to the untreated cells. Scaffolds loaded with higher concentrations of Manuka honey and Aloe vera enhanced cell migration, whereas eucalyptus essential oil had an important role in the antibacterial efficacy against both positive and negative bacteria [132].

The use of multifunctional materials has been also investigated in this area. Wu and coworkers (2021) developed 3D printed hydrogels loaded with tea polyphenols, which have antioxidant, antibiotic, antitumor, radiation resistance, wound healing properties and immunity enhancement properties [183]. First, hydrogel composed of natural polymers (GelMA and sodium alginate) were printed by SSE in different shapes: triangle, rectangle, and hexagon. Thereafter, they were soaked in a tea polyphenols solution, which improved the mechanical properties of the printed hydrogels. The GelMA hydrogels loaded with the tea polyphenols showed good antioxidant properties in scavenging free radicals and antibacterial activity against E. coli and S. aureus, demonstrating their potential to inhibit the growth of gram positive and negative bacteria. Furthermore, in vivo studies in rats highlighted the ability of these hydrogels to accelerate wound closure and skin regeneration [154]. A similar strategy was explored by another research group using Satureja cuneifolia (SC) extract, which has antioxidant, antidiabetic, and antimicrobial properties, among others. The SC extract was loaded into a 3D printed scaffold produced by SSE and composed of sodium alginate and polyethylene glycol to design wound dressing materials with antimicrobial activity for the diabetic healing process. The data reported by the authors demonstrated good printability of the hydrogel affording scaffolds with the desired porosity and excellent antibacterial effect, being, therefore, promising candidates for diabetic wound healing [131].

As discussed above, additive manufacturing products containing natural compounds have recently gained interest for wound healing purposes. Regarding all the studies presented here, promising new therapies have been investigated for the treatment of skin injuries, which will be more effective and less harmful once these novel approaches are properly regulated and translated into the clinic.

Based on all the studies presented in this section, we spotted the potential of delivery of active ingredients for topical skin applications by 3D printed dosage forms. 3D printing can promote the development of complex structures that cover a wide range of drug release possibilities and with multifunctional purposes in a simple, but versatile way. It represents a smart and advanced manufacturing technology to produce customized therapies that opens a whole new branch of design possibilities in terms of composition, structure, size, dose, texture, pore size, swelling characteristics, among other factors that can have a significant impact on the efficiency of the skin diseases therapies or their better patient’s acceptance.

Despite these positive outcomes, some critical points must be considered during the printing process, which is intrinsic to the methodology used, such as the risks of drug degradation due to high temperatures, in the case of FDM; or even due to the light source, in the case of SLA and the compatibility among all the formulation components. The influence of the active ingredient on the ink’s rheology for SSE printing should be also well-characterized to facilitate the development of topical drug delivery systems.

It is important to mention that most of the studies presented here focused on the treatment of wound healing. However, given their positive outcomes in the field and the proven advantages over other dosage forms, the development of 3D printing skin delivery devices should be extended to other important skin diseases. Like all emergent areas, there are still some important challenges and scientific questions to be addressed, such as the permeation and penetration of actives into the skin from 3D structures, since very few studies discussed here have been performed this type of evaluation. Therefore, some efforts should be made to assess the permeation and ensure the safety and effectiveness of these treatments.

## 6. Regulation, Safety Considerations and Perspectives

3D printing and topical skin applications are two promising approaches for healthcare solutions. Their alliance is a very new perspective that can contribute to more personalized and complex drug delivery systems and wound dressing materials. From a pharmaceutical point of view, the use of 3D printing for the development of skin drug delivery systems can provide many advantages that were highlighted in this review. Different polymers have been used in the development of topical products. Synthetic hydrophobic materials, such as PCL and PLGA, are preferred for wound protection and for an external composition of dressings as they are more rigid, have low gas permeation and prevent bacterial invasion [90,94]. On the other, natural polymers, such as alginate, have a high capacity for absorbing exudates, making this material a good alternative to keep in contact with the wound and to accelerate cell proliferation [90]. The hydrogels composed of natural polymers also help to keep a moist microenvironment, which is favorable for wound healing. In addition, some natural polymers, as chitosan, have intrinsic properties, including antimicrobial and bioadhesive activity, providing the development of multifunctional systems [142]. Besides, there is a need for multidisciplinary research aiming for prosperous and successful product development.

Although several positive points of 3D printing have been pointed out, it is worth noting that there are still challenges and limitations to the implementation of this technology. The development of ideal 3D printers, which can overcome completely current limitations requires collaborative work between engineers, scientists, excipients suppliers and pharmaceutical regulators [49]. These challenges include the standardization of printers and printing techniques, safe materials and polymers depending on the administration route, development of non-destructive analytical methods, regulatory aspects, and possible unintended consequences [17,184]. Regulatory issues remain one of the biggest challenges of 3D printing. US FDA regulations are proposed to mass production and standardization of batches, which cannot be easily translated to 3D manufactured personalized products [185]. Based on the necessity of regulatory considerations, in 2017 a technical guidance was published by FDA, called: “Technical Considerations for Additive Manufactured Medical Devices” [186]. This guidance provided the initial thinking on technical considerations, and it is clear that some parameters will have to be ensured aiming quality control, such as hardware, software, materials and supplies, patient-matched device design, operator training, quality control protocols, sterilization issues, the critical point for intermediate products (such as filaments), post-printing, process validation, among others [49,184,186]. However, many questions are still open, and it is expected that there will be additional regulations in the near future. It is important that regulatory agencies, pharmaceutical companies, and academy researchers stay ahead of these issues and discuss them, therefore, the regulatory aspects can evolve and target even more 3D printing products in the current market. In addition, long-term stability studies must be carried out for the 3D printed products [10].

In addition, most of the commercialized 3D printers do not fulfill good manufacturing practice requirements, being an important factor to be overcome, although most of them are low cost and portable, which allows the implementation of this technology in pharmacies, pharmaceutical companies and hospitals for on-demand personalized products. Of course, the one-demand approach will not compete with the mass production by the pharmaceutical industry, as the manufacture of 3D printed devices can take hours while traditional formulations, such as tablets, can be produced by the thousands in just a few minutes [187]. These concepts are completely different. On the other hand, a strategy to speed up the large-scale production of 3D printed dosage forms has been discussed based on 3D printer farms. This strategy combines the use of many 3D printers in the same room, producing the same product with control of the production [188]. On the other hand, the on-demand manufacture of pharmaceuticals at pharmacies, hospitals and health services still needs many discussion rounds regarding some ethical, stability and quality control concerns [189,190]. Even so, as discussed here, printing parameters (e.g., shape, size, pores) can affect the performance of 3D printed products, hence it is fundamental that an assay is capable of predicting drug content and performance of these products [191].

Regarding skin products requirements, we highlighted that there is a gap in the permeation and penetration studies, which are crucial to ensure the safety of these products. Additionally, quantitative analysis of drug distribution in the skin is critical to fully understand the interaction between the drug, delivery system and the skin [35,192]. Alternative methods for characterization of skin penetration/permeation studies are encouraged and nowadays there is a great range of technologies, such as static diffusion cells, the use of ex-vivo skin membranes, artificial membranes, reconstructed skin models, mathematical models, among others [192].

Moreover, depending on the 3D printed product skin application, sterilization can be a crucial point for the full performance of the product, such as wound dressing, for example [193]. Although some studies have signalized a sterilization phase prior to antibacterial, cell or in vivo studies, there is still a lack of discussion about the most adequate sterilization methods. In confluence with the Culmone and coworkers (2019) findings, debris remaining after sterilization is still a gap to be studied, even though this point is almost never considered [194].

There is a wide range of sterilization techniques, such as moist heat by autoclave, ultraviolet radiation (UV), filtration, ethylene oxide gas, hydrogen peroxide gas plasma, gamma irradiation, among others [193,195]. Autoclave is a non-toxic technique; however, it uses high temperatures during sterilization (121 ºC and 132 ºC), which can be critical to certain polymeric materials. Ethylene oxide gas may be an interesting approach for sterilization, once it is a low-temperature method. This technology has the disadvantage of being a toxic gas, and so, FDA recommendation is to use it only if the materials are heat/or moisture sensitive, and therefore, cannot be steam sterilized. UV radiation has potential application, however, its effectiveness relies on many parameters, such as the presence of organic matter, temperature, microorganism, UV intensity and wavelength, among others. Filtration sterilization is a very useful technique for fluids even so is not an FDA-cleared sterilization method. This technology uses membrane pore size smaller than the bacteria (e.g., 0.22 um) and is used for thermolabile pharmaceutical fluids that cannot be sterilized by other methods [195]. However, filter sterilization use is limited, and cannot be applied for semi-solids and solids, being even more difficult for topical 3D printed products. Another possibility is the preparation under aseptic conditions, based on initial and separate sterilization of each constituent and container (e.g., filtration, heat, etc.) and preparation in a sterile environment. This alternative involves more variables and a stricter control since there is no final sterilization process [196]. However, it can be an option for 3D printing if it is difficult to find a suitable sterilization technique.

Gamma irradiation is a widely used method for sterilization, however, it can affect structural properties on polymer medical devices and carry potentially adverse effects [197]. Nevertheless, Navarro et al., (2020) evidenced the influence of gamma sterilization on keratin hydrogels and based on their data, gamma penetration is more vulnerable for thinner samples, which results in breakage of the crosslinked network. Gamma irradiation may also reduce the 3D printed sample thickness after the sterilization process [135]. The geometry of the scaffolds plays an important role in dermal wound healing, as they should fit the wound site, accommodate the thickness of the wounds [135]. Therefore, it is of major importance to carefully control the post-printing manufacturing parameters, such as the sterilization process that may affect the size, thickness, or other properties of the dressing.

In general, most of the studies discussed here intend to prove some concepts about the application of 3D printing technologies for topical administration, being at a prototyping phase, explaining the lack of sterilization concerning. Meanwhile, we encourage researchers to consider sterilization as an important factor in the development of 3D printed products for skin use, mainly if they are designed to induce wound healing. Furthermore, it is noteworthy that 88% of the articles presented here designed products for wound healing, which is of paramount importance. However, we would also like to highlight that there is a multitude of other dermatological diseases that can be explored in the future and that would greatly benefit from 3D printing.

Lastly, it is worth mentioning that some researchers have coined a new term: 4-D printing, as one step further. This new concept takes 3D printing to the next level and aims to produce highly accurate 3D structures that can experiment changes over the fourth dimension, time [198]. The main requirements to carry out this new approach are those related to the smart materials responding to external stimuli (moisture levels, temperature, pH, magnetic field, etc.) and triggering the evolution of the printed device, such as shape memory polymers [199]. We guess we will be able to discuss the application of this concept also in the development of skin delivery devices in the following years.

## 7. Conclusions

Herein, we demonstrated the feasibility and advantages of introducing 3D printing technology in the development of novel topical skin products. It is remarkable that it is a very modern subject, but with exponential growth in the last years. 3D printing allows the manipulation and customization of fundamental dosage form parameters for a more effective and comfortable therapy for the patient, showing excellent opportunities in the area. This emerging technology allows the personalization of size, dose, pore structure, swelling properties, disintegration, and drug release profile of pharmaceutical dosage forms, along with the potential to produce high complexity devices at cost-effective production. 3D printing can include drugs, metals, proteins, and natural compounds in the printed products for topical applications. In addition, there is an excellent field for customizing dressings according to individual wound shape and needs. The confluence of this 3D printing and topical drugs has undeniable advantages to improve patient life quality and treatment efficacy. This review paves the way for further studies to broaden the application of 3D printing in the development of topical skin products, such as drug delivery systems or dressings with advanced and multifunctional properties.

## Figures and Tables

**Table 1:** 3D-printed plain dressings.

3D Printing Technique	Main Material	Application	Printed Form	Reference
SSE	Nanocellulose	Wound dressing	Grid	[79]
SSE	Alginate and gelatin	Recover full-thickness skin wounds	Scaffold	[80]
Jet dispensing (micro-jetting system)	Alginate	Robotically deposited bioprinting hydrogel directly onto moving human hand	Hydrogel	[81]
SSE	Gellan gum	Wound dressing or cartilage replacement	Scaffold	[82]
n/d	ABS	Burn wound healing	Artificial skin	[83]
Extrusion based	Chitosan	Skin tissue regeneration and diabetic wound healing	Scaffold	[84]
SSE	PEGMA-sodium acrylate-PEGDA and kaolin	Absorbent and hemostatic wound dressing	Dressing	[85]
SSE	Nanocellulose	Wound healing, regeneration and tissue repair	Hydrogel scaffold	[86]
SSE	Alginate and nanocellulose	Wound dressing devices	Grid wound dressing	[87]
SSE	Xylorhamno uronic acid and gelatin	Skin repair and wound healing	Hydrogel scaffold	[88]
SSE	Alginate and cellulose nanofibrils	Wound dressing	Hydrogel dressing	[89]
SSE	Chitosan and sodium alginate	Skin regeneration	Skin construct/membrane	[90]
SSE	PVA and sodium alginate	Bilayered polymeric carriers for wound care	Mats	[91]
Cryogenic free-form extrusion	Decellularized small intestinal submucosa	Skin tissue engineering	Scaffold	[92]
SSE	Nanocellulose and GelMA	Wound healing	Scaffold	[93]
EHD e SSE	PLGA and sodium alginate	Deep wound healing	Membrane scaffold	[94]
SSE	Gelatin and cellulose nanocrystals decorated with nitrogen-doped carbon dots	Skin wounds	Hydrogel dressing	[95]
Extrusion-based direct-write	PCL and functionalized polyesters	Wound healing	Scaffold dressings	[96]
Hybrid extrusion	Alginate, CMC and PCL	Tissue engineering and wound dressing	Scaffold	[97]
SSE	GelMA	Wound healing and skin repair	Scaffold	[98]
FDM	PVA, PU and PCL	Wound dressing	3D lattices	[99]
n/d	CMC and ε-polylysine	Skin wound repair	Hydrogel dressing	[100]
Cryogenic extrusion-based	Chitosan and bioactive glass	Wound healing	Scaffold	[101]

n/d, not clearly defined (according to the description described by the authors, it was not possible to categorize it in this review); ABS, acrylonitrile butadiene styrene; CMC, carboxymethyl cellulose; EHD, electrohydrodynamic; FDM, fused deposition modeling; GelMA, gelatin methacrylate; PCL, polycaprolactone; PEGDA, poly(ethylene glycol) diacrylate; PEGMA, poly(ethylene glycol) methacrylate; PLGA, poly (lactic-co-glycolic acid); PU, polyurethane; PVA; Poly(vinyl alcohol); SSE, semisolid extrusion;.

**Table 2:** 3D-printed products for topical delivery of active ingredients.

3D Printing Technique	Main Materials	Active Ingredient	Active Incorporation Method	Application	Printed Form	Reference
FDM	NF, FLPA and PCL.	Salicylic acid	Preprint: solvent casting prior filament fabrication by hot melt extrusion	Anti-acne personalized patch	Patch/mask	[113]
SLA	PEGDA and PEG		Preprint: dispersion in the polymer			
DLP	3DM-Castable resin	Diclofenac	*	Trigger finger treatment	Microneedle	[116]
FDM	PCL	Zinc, copper and silver	Preprint: solvent casting prior filament fabrication by hot melt extrusion	Antimicrobial personalized wound dressings	Dressing	[112]
EHD	PCL and PVP	Tetracycline	Preprint: blended in the ink	Tetracycline loaded patches for personalized drug delivery	Patch	[117]
SSE	Alginate and CMC	Sodium diclofenac and lidocaine	Preprint: blended in the ink	Pain-relieving scaffolds for wound healing applications	Scaffold	[9]
n/d	PLLA and gelatin	Platelet rich fibrinogen	Postprint: blended in the 3D printing powder	3D printed scaffolds for chronic wounds	Scaffold	[118]
SSE	PEGDA, mineral oil and Kolliphor^®^ P188	Gallium maltolate	Postprint: soaking method	Hydrogel wound dressing with antimicrobial agent	Hydrogel dressing	[119]
FDM	Lignin, PLA and castor oil	Tetracycline	Preprint: blended with polymers prior hot melt extrusion for filament fabrication	Drug loaded meshes with antioxidant activity for wound dressing application	Mesh	[120]
		Curcumin	*			
Jet dispensing	Chitosan	Fluorescein sodium	Preprint: blended in the ink	Films for wound healing applications	Film	[121]
MEW	PCL	Milk proteins (whey protein and lactoferrin)	Preprint: blended in the ink	Milk proteins loaded scaffold for deep skin tissue regeneration	Scaffold	[122]
SSE	Chitosan and pectin	Lidocaine hydrochloride	Preprint: blended in the ink	Hydrogel dressing loaded with local anesthetic for wound dressing	Hydrogel scaffold	[123]
SSE	PDMS	Silver nanoparticles	Preprint: blended in the ink	Wound dressing with silver nanoparticles and oil infusion for wound healing activity	Dressing membrane	[124]
SSE	GeLMA	PDGF-BB	Preprint: blended in the ink	Skin-inspired bilayer scaffold for diabetic wound healing	Scaffold	[125]
		Silver nanoparticles	Postprint: impregnation			
FDM	PLA	Silver nanoparticles	*	Antibacterial Superporous Hydrogels Wound Dressing	Porous dressing templates	[126]
Melt extrusion	PCL-PPSu block copolymers	Silver	Preprint: polymer impregnation prior printing	Scaffold with antimicrobial properties for skin tissue engineering	Scaffold	[127]
SSE	Pectin and manuka honey	Chitosan and β-cyclodextrin/propolis extract inclusion complexes	Preprint: blended in the ink	Bio-active dressing patch for ulcers and wound healing applications	Patch	[128]
SSE	Sodium alginate	Propolis	Preprint: blended in the ink	Propolis-sodium alginate scaffolds for wound healing applications	Scaffold	[129]
SSE	Alginate and starch	Rhodamine B	Preprint: blended in the ink	Topical hydrogel patch for drug delivery	Hydrogel patch	[111]
FDM	Resin VeroClear, and Tango black	BSA, VEGF, cefazolin	*	Wound bandage with miniaturized needle array for wireless actively delivery of drugs	Miniaturized needle array	[130]
SSE	Sodium alginate and PEG	Satureja cuneifolia extract	Preprint: blended in the ink	3D printed loaded scaffold for diabetic wound treatment	Scaffold	[131]
SSE	Alginate and methylcellulose	Manuka honey, aloe vera gel and eucalyptus essential oil	Preprint: blended in the ink	Hydrogel loaded with bioactive components for wound healing applications	Hydrogel film	[132]
DLP	3DM-Castable resin	Acetyl-hexapeptide-3	*	Microneedle patch with different geometries and curvature for anti-wrinkle peptide delivery	Microneedle patch	[133]
SSE	PLGA	Mupirocin	Preprint: blended in the ink	3D printed scaffold to cover piercing studs for preventing piercing infections	Scaffold	[134]
DLP	Keratin	Halofuginone	Postprint: impregnation	Keratin loaded scaffold for burn wounds healing	Scaffold	[135]
SSE	Sodium polyacrylate	Cryptotanshinone niosome	Preprint: blended in the ink	Cryptotanshinone loaded niosome for topical delivery in acne treatment	Hydrogel	[136]
SSE	Cellulose nanocrystals and chitosan methacrylamide	VEGF, BSA, silver nanoparticles and gentamicin	Preprint: blended in the ink	3D-printed multifunctional wound dressing	Hydrogel	[137]
EHD	Bacterial cellulose and PCL	amoxicillin, ampicillin, and kanamycin	Preprint: blended in the ink	Antibiotic patches for local transdermal delivery in wound healing applications	Patch	[138]
FDM	PLA	Cu-CDs	Postprint: soaking method	Nanocomposite containing PLA/HA/chitosan/Cu-CDs/rosmarinic acid for wound healing applications	Scaffold	[139]
N/D	Chitosan and alginate	Epidermal Growth Factor	Postprint: solution was embedded or directly dropped in the scaffolds	Multifunctional dressings for local release of therapeutic adjuncts	Scaffold	[140]
Material jetting	VeroClear RGD810 and TangoBlack FLX973 resins	Fetal bovine serum, VEGF and rhodamine B	Postprint: by filling the microneedles	Microneedle arrays for drug delivery applications	Microneedles patch	[141]
Freeze-deposition	Chitosan	α-tocopherol	Preprint: blended in the ink	Active dressings for chronically infected wounds	Scaffold	[142]
Cryogenic extrusion based	Mesoporous bioglass, sodium alginate and decellularized small intestinal submucosa	Exosomes	Postprint: exosome solution was wrapped in the scaffold	Hydrogel scaffold for diabetic wound healing	Hydrogel scaffold	[143]
FDM	PCL	Gold nanoparticles	Postprint: soaking method	Scaffold loaded with gold nanoparticles for skin regeneration	Scaffold	[144]
DLP	Vinyl pyrrolidone and PEGDA	Acetyl-hexapeptide-3	Preprint: blended in the polymers resin	Personalized microneedle patch for anti-wrinkle peptide delivery	Microneedle patch	[145]
SSE	Polyacrylamine and gelatin	Silver nanoparticles	Preprint: blended in the ink	Printable inks with antibacterial and anti-UV properties	Scaffolds	[146]
SSE	Starch and N, O-carboxymethyl chitosan	Mupirocin	Preprint: blended in the ink	Hybrid biomaterial ink for 3D printed wound dressings	Scaffold	[147]
SSE	Methylcellulose, alginate, PNIPAAm	Octenisept^®^ (octenidine dihydrochloride and 2-phenoxyethanol)	Postprint: soaking method	Thermoresponsive 3D printed hydrogel loaded with antimicrobial agent for wound healing applications	Hydrogel dressing	[148]
FDM	VeroClear resin	VEGF	*	Miniaturized needle array for VEGF intradermal delivery for wound healing application.	Miniaturized needle array	[149]
SSE	Alginate	Bacteriophage nanoparticles	Preprint: blended in the ink	Bacteriophage-based antibacterial wound dressing	Hydrogel dressing	[150]
SSE	GelMA and gelatin	VEGF and ZnO	Preprint: blended in the ink	Smart wound scaffold with antibacterial active	Hydrogel patch	[151]
Hot melt extrusion-based	PLLA	Neomycin	Postprint: soaking method	Neomycin loaded mats for wound healing applications	Mats	[152]
SSE	Chitosan methacrylate	Lidocaine hydrochloride and levofloxacin	Preprint: blended in the ink	Wound dressing for thermal burns	Hydrogel dressing	[153]
SSE	Sodium alginate and GelMA	Tea polyphenols	Postprint: soaking method	Hydrogel with antibacterial and antioxidant activities for wound healing and treating	Hydrogel scaffolds	[154]

* Active ingredients are not incorporated into the 3D printed product itself; n/d, not clearly defined (according to the description described by the authors, it was not possible to categorize it in this review); BSA, bovine serum albumin; CMC, carboxymethyl cellulose; Cu-CDs, copper carbon dots; DLP, digital light processing; EHD, electrohydrodynamic; FDM, fused deposition modeling; FLPA, Flex ecoPLA™; GelMA, gelatin methacrylate; HME, hot melt extrusion; MEW, melt-electrowriting; NF, Ninja Flex^®^; PCL, polycaprolactone; PDGF-BB, platelet derived growth factor-BB; PEG, poly(ethylene glycol); PEGDA, poly(ethylene glycol) diacrylate; PEGMA, poly(ethylene glycol) methacrylate; PLA, polylactic acid; PLLA, poly-l-lactic acid; PMDS, polydimethylsiloxane; PNIPAAm, poly(N-isopropylacrylamide); PPSu, poly(propylene succinate); PVP, polyvinylpyrrolidone; SLA, stereolithography; SSE, semisolid extrusion; VEGF, vascular endothelial growth factor; ZnO, zinc oxide.
